# Cytokeratin19-2g2, a Novel Fragment of Cytokeratin19 in Serum, Indicating a More Invasive Behavior and Worse Prognosis in Breast Cancer Patients

**DOI:** 10.1371/journal.pone.0057092

**Published:** 2013-02-28

**Authors:** Yanan Kong, Junye Wang, Wanli Liu, Qiaolun Chen, Juan Yang, Weidong Wei, Mingqing Wu, Lu Yang, Xinhua Xie, Ning Lv, Jiaoli Guo, Laisheng Li, Jie Gao, Xiaoming Xie, Shuqin Dai

**Affiliations:** 1 Department of Breast Oncology, Sun Yat-Sen University Cancer Center, Guangzhou, Guangdong, People’s Republic of China; 2 State Key Laboratory of Oncology in South China, Sun Yat-Sen University Cancer Center, Guangzhou, Guangdong, People’s Republic of China; 3 Department of Medical Examination, Sun Yat-Sen University Cancer Center, Guangzhou, Guangdong, People’s Republic of China; 4 Department of Chest Oncology, Sun Yat-Sen University Cancer Center, Guangzhou, Guangdong, People’s Republic of China; Dartmouth, United States of America

## Abstract

**Background:**

Various studies have been searching for new tumor biomarkers for breast cancer for years. However, so far, few markers have been proved clinically useful except CA153. Based on knowledge that most adenocarcinomas including breast carcinoma expressed Cytokeratin19, the authors studied CK19-2G2,a novel fragment of cytokeratin19 shedding into serum in breast cancer patients.

**Patients and Methods:**

The serum samples of four hundred and seventeen patients including three hundred and three (fifty-four DCIS and two hundred and forty-nine stage I-III) PBC patients and one hundred and fourteen MBC patients, eighty-one healthy controls and twenty-one breast benign disease patients were provided for measurement of CK19-2G2, CEA and CA153.The correlation between clinicopathological characters, prognosis and CK19-2G2 levels was further studied.

**Results:**

The serum CK19-2G2 levels in breast cancer patients were significantly higher than that in healthy and benign controls. For breast cancer patients, CK19-2G2 levels in MBC were significantly higher than that in PBC patients. The sensitivities of CK19-2G2 for breast carcinoma are as high as CEA and CA153, and up to 71% in MBC patients. Serum CK19-2G2 levels (≥2 mU/mL) were associated with pathological stages, tumor size (≥2 cm), lymph node involvement, and HER2 status. Multivariate analysis revealed that high serum CK19-2G2 level was an independent factor for relapse (*P = *0.029) and death (*P = *0.040) in breast cancer patients.

**Conclusion:**

Serum CK19-2G2 may be an independent indicator for prognosis and a candidate marker for monitoring metastasis in breast cancer.

## Introduction

Breast cancer is one of the most common malignancies and the leading cause of mortality in women in western countries and in China. According to American Cancer Society, there were about 230,480 estimated new cases and 39,520 deaths caused by breast cancer in 2010 in the United States, that account for 30% of all new malignant cases and 15% of death caused by cancers respectively [1]. Over the past few decades, new techniques and methods in diagnosis and treatment have been developed, leading to increased survival of breast cancer patients. However, many breast cancers still can not be detected at early stage. Currently, a variety of biological tumor markers are studied to diagnose these early diseases, monitor recurrence or metastasis in treated patients, and predict response or resistance to therapies. Clinically available tumor markers for breast cancer mainly include carcinoembryonic antigen (CEA) and CA153. CEA, a cell-surface glycoprotein that is expressed in normal mucosal cells and overexpressed in a wide variety of adenocarcinomas, including colon, rectum, breast, pancreas, and lung, has been recognized as one of the most useful tumor markers in clinical practice [2]. CA153, a mucin-like membrane glycoprotein belonging to a large family of glycoproteins encoded by the *MUC 1* gene [3], has been considered as a representative tumor marker for breast cancer. However, current American Society of Clinical Oncology and National Comprehensive Cancer Network guidelines do not recommend CA 153 or CEA to be used in screening, diagnosis, staging and surveillance of recurrences after primary treatment or evaluating response to treatment due to insufficient present data [4]. Cytokeratin-19 (CK-19), a cytoskeletal component expressed in normal and cancerous epithelial cells, has been demonstrated present in both patients with early-stage and metastatic breast cancer [5], [6]. Recently, the prognostic significance of CK-19 mRNA-positive CTCs (circulating tumor cells) in patients with breast cancer has been reported [7]–[10]. Ck19-2G2, a novel one of cytokeratin 19 fragments shed into circulation, which was identified both by CK192G2 and CK19 5H2 antibody, has been considered as one of the most sensitive tumor marker for lung carcinoma, even superior to CYFRA21-1, another CK19 fragment shed into blood in lung cancer patients [11]. So far, there has not been any study about CK19-2G2 in breast cancer. We compared serum cytoketatin19-2G2 levels in breast cancer with that in healthy and benign controls respectively, explore the correlation of serum CK19-2G2 with CEA, CA153 and clinicopathological characteristics, and finally evaluate the prognostic value of serum CK19-2G2 in breast cancer patients.

## Patients and Methods

Four hundred and seventeen breast cancer patients including fifty-four patients with ductal carcinoma in situ(DCIS), two hundred and forty-nine primary breast cancer (PBC) patients with stage 1-III, one hundred and fourteen patients with metastatic breast cancer (MBC) in Sun Yat-Sen University Cancer Center in southeast of China were enrolled in this study. The mean age was 48.5 years and the standard deviation (SD) was 10.9 years. Eighty-one healthy and twenty-one women with breast benign diseases were also included as controls. Serum samples of patients with PBC were obtained before surgeries and with MBC were acquired at the time of diagnosis. For primary breast cancer patients, the survival period was defined as the time between the day when the serum samples were taken and December 31, 2010 for all living patients, or until the day of death. Our study was permitted by Sun Yat-sen University Cancer Center Ethnic Committee (20120013). All participants provide their written informed consent (as outlined in PLOS consent form) to participate in this study and to publish these case details.

### Serum CK19-2G2, CEA, CA153 Assays

Serum samples were obtained from the department of breast oncology in our cancer center, which were collected at the time of cancer diagnosis and stored at −80°C. The measurement of CK19-2G2 was performed in a two-step sandwich enzyme immunoassay using Diagnostic Kit of Cytokeratin-19-2G2 Fragments with Chemiluminescence Quantitative Immunoassay(CLIA). (Tongsheng Times, Peking, China) performed on an automated BHP9504 analyzer (Tongsheng Times, Peking, China).The kit was comprised of two monoclonal antibodies: CK19-2G2 (CK19 aa 375–400) and CK19-5H2 (CK19 aa 325–350). In addition, we measured CEA and CA 153 levels in serum, which currently are taken as useful tumor markers for breast cancer. CEA and CA 153 were assessed both by chemiluminescence immunoassay using a commercially available kit (Cobas; Roche, US). According to the producer’s instruction, the cutoff value of CK19-2G2 was determined to be 2.0 mU/mL. The cutoff values of CEA and CA 153 recommended by the manufacturers were 5.0 ng/mL and 30 U/mL, respectively.

### Clinicopathological Characteristics and Follow-up

Clinicopathological characteristics of stage I-III PBC patients including age, menopausal status, tumor size, lymph node status, stage, ER status, PR status and adjuvant therapy were shown in Table 1. Pathological stage of tumor was classified according to the TNM staging system (American Joint Committee on Cancer classification). Physical examination, image examination (ultrasound of contralateral breast, liver, mammography, chest X-ray, ECT and further image modalities such as diagnostic CT if indicated) and blood tests for CEA and CA153 were carried out during follow-up (every 3 months for the first 2 years, every 6 months for the next 3 years and one year after 5 years). First appearance of a new disease in local area, contralateral breast, distant organs or in combination of these was defined as disease progression.

**Table 1:** PBC patients and tumor clinicopathological viariables.

Characteristics	No. of patients	%
Age (years)		
≤35	29	11.6
>35	220	88.4
Menopausal status		
Premenopausal	143	57.4
Postmenopausal	106	42.6
Stage		
I	3	1.2
II	35	14.1
III	133	53.4
Undetermined	59	23.7
Tumor size (cm)		
≤2	70	28.1
>2	160	64.3
Undetermined	19	7.6
Lymphonodes status		
Negative	119	47.8
Positive	126	50.6
Unknown	4	1.6
ER status		
Negative	93	37.3
Positive	152	61.1
Unknown	4	1.6
PR status		
Negative	111	44.6
Positive	134	53.8
Unknown	4	1.6
Tissue HER2(IHC/FISH)		
Negative	183	73.5
Positive^a^	61	24.5
Unknown	5	2.0
Adjuvant systematic treatment		
Adjuvant CT	153	61.4
Endocrine therapy	90	36.1
Only endocrine therapy	9/90	10.0
Endocrine therapy after CT	81/90	90.0

a IHC 3+, IHC2+ and FISH amplified.

ER, estrogen receptor; PR, progesterone receptor; CT, chemotherapy; HER2, human epidermal growth factor receptor-2; ECD, extracellular domain; IHC, immunohistochemistry; FISH, fluorescence in situ hybridization.

### Statistical Analysis

The Anova test was used for a comparison among more than two groups and the Mann–Whitney U test for a comparison between two independent groups. The Kruskal–Wallis one-way analysis was used for a multiple comparison test. Chi-Square test and correction for continuity were used to compare the sensitivity of serum CK19-2G2 and other tumor markers, and evaluate the relationship with clinicopathological characteristics in all 2_2 tables. Disease Free Survival (DFS) was calculated as time from surgery to disease progression or death, whichever occurred first. Patients who were alive and disease free were censored at the date of last follow-up visit. Overall Survival (OS) was defined from surgery to death for any cause, and patients who were alive were censored at date of last follow-up visit. Survival curves were obtained by Kaplan-Meier and estimated values were examined by the log rank test.We used Cox proportional hazards models to evaluate the impact of serum CK19-2G2 and clinicopathological characteristics on DFS and OS. Univariate and multivariate analysis were both used in proportional hazards. Hazard ratio (HR) with 95% confidence interval (CI) was used to describe this. Statistical significance was taken as *P*≥0.05.

Serum CK19-2G2 levels were bifurcated at 2.0 mU/ml. All the data were analyzed using SPSS for Windows Release 13.0 and all *P* values were significant at a two-sided test. Appropriate comparison method was used according to the distribution of data after Test of Normality.

## Results

### Serum CK19-2G2 Levels in Breast Cancer Patients, Benign and Healthy Controls

**Figure 1:**
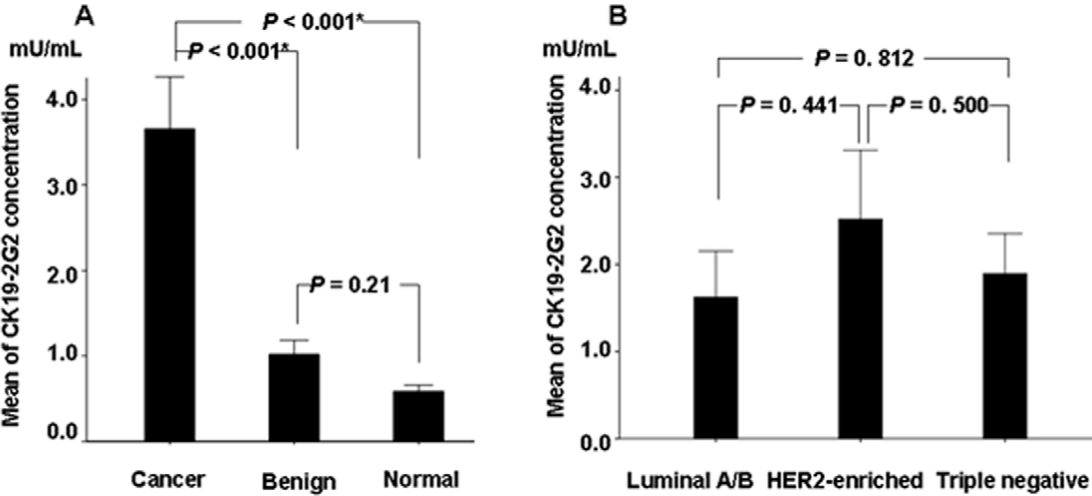
Serum CK19-2G2 levels in breast cancer patients. (**A**) The Mean of serum CK19-2G2 levels in healthy, benign, BC group. Serum CK19-2G2 levels in breast cancer patients are higher than those in the healthy and benign controls respectively.(BC *vs Benign, P*<0.001; BC *vs Normal, P*<0.001) (Mann-Whitney *U* test).Error bars are referring to SE(Standard Error). (**B**) The Mean of serum CK19-2G2 levels in breast cancer patients with LuminalA/B, HER2-enriched and Triple negative type. There was no difference among the three groups.Error bars are referring to SE(Standard Error).

There was a difference in the distribution of serum CK19-2G2 levels among breast cancer patients, Benign and Healthy controls.(Figure1A). Serum CK19-2G2 levels in breast cancer patients (Mean = 3.66 mU/ml, SE = 0.61 mU/ml) were significantly higher than those of the healthy group (Mean = 0.58 mU/ml, SE = 0.07 mU/ml) and the benign group (Mean = 1.01 mU/ml, SE = 0.17 mU/ml). No increased serum CK19-2G2 levels were observed in both healthy (Range 0–1.96 mU/ml) and benign groups (Range 0–1.98 mU/ml) and there was no difference between the healthy and benign group(*P* = 0.21). Therefore, the specificity of serum CK19-2G2 for breast carcinoma was 100% (specificity: samples without breast carcinoma with negative tests/all samples without breast carcinoma tested).

**Figure 2:**
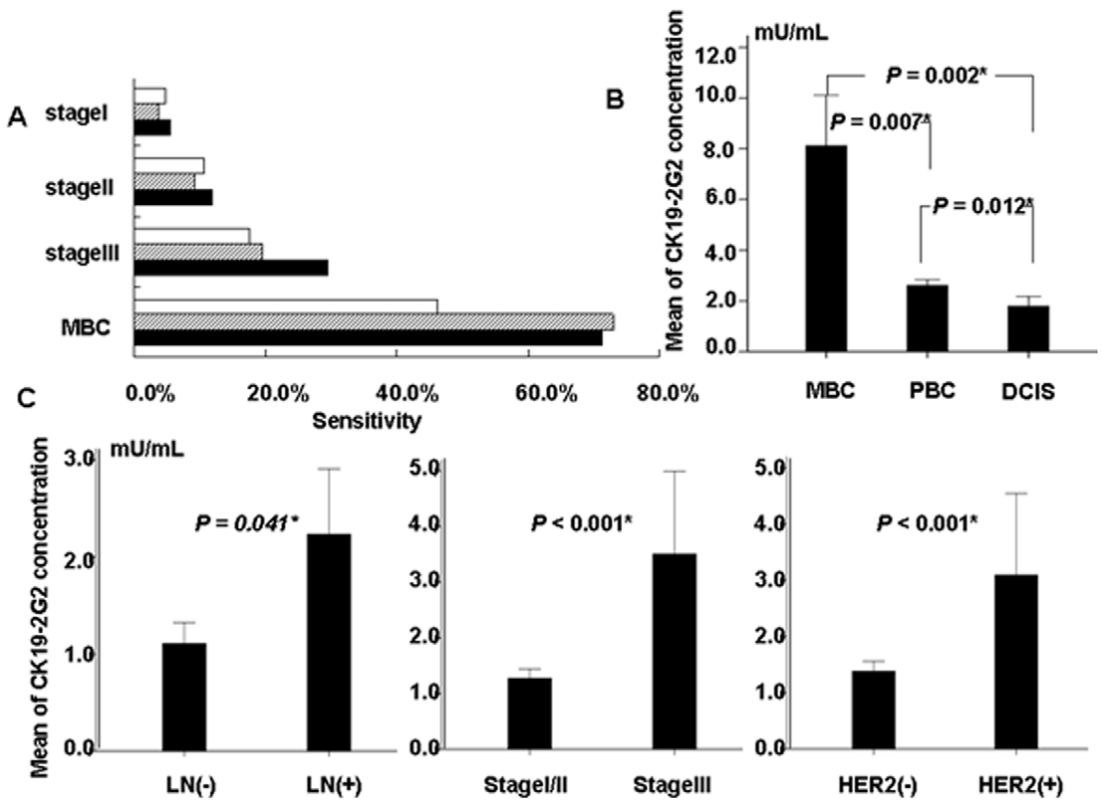
The sensitivity of CEA, CA153 and CK19-2G2 for PBC and MBC. (**A**) CK19-2G2 is superior to CEA and CA153 in stage II and III breast cancer patients and as sensitive as CA153 in MBC patients. The blank bar: CEA; the gray bar: CA153; the black bar: CK19-2G2 (**B**) The distribution of CK19-2G2 in MBC, PBC and DCIS. Serum CK19-2G2 levels in MBC are higher than those in the PBC and DCIS respectively(MBC *vs PBC, P = *0.007; MBC *vs DCIS, P = *0.002). Serum CK19-2G2 levels in PBC are higher than those in DCIS (*P = *0.012). Error bars are referring to SE(Standard Error). (**C**) The distribution of CK19-2G2 in breast cancer patients with different stages, lymph nodes status and HER2 status. Patients with positive lymph nodes, stage III and HER2 positive status had higher serum CK19-2G2 levels than those with negative lymph nodes, stage I/II and HER2 negative status. Error bars are referring to SE(Standard Error).

According to ER, PR, HER2, Ki67 status, breast cancer was classified into four types in clinical practice: LuminalA, LuminalB, HER2-enriched and Triple negative. We tested the serum CK19-2G2 levels in different type in PBC patients with stage I-III, and found that CK19-2G2 levels in HER2 enriched and Triple negative type were higher than that in LuminalA/B type, however, there was no statistical difference among the three groups (Figure 1B). The serum CK19-9 levels of breast cancer patients with stage III (3.51±11.29 mU/mL) were significantly higher than those with stage II/I (1.27±2.13 mU/mL) (P<0.001) (Figure 2B). When the cutoff value was defined to be 2.0 mU/mL, the percent of abnormal serum CK19-2G2 (sensitivities) for patients with Stage I, II, III and MBC were 5.4%, 11.9%, 29.5% and 71% respectively (Figure 2A).

### Comparison and Correlation between Serum CK19-2G2 and CEA, CA 153

The sensitivity for serum CK19-2G2, CEA and CA153 in diagnosis of primary breast cancer was 12.2%, 11.9% and 12.6% respectively. Figure 2A shows that the sensitivity of CEA, CA153 and CK19-2G2 in patients with Stage II–III. CK19-2G2 levels were observed elevated in 71% MBC patients, that is almost as sensitive as CA153. Chi-Square test was used to compare the sensitivity of the three markers respectively and we found that there is no statistical difference between CK19-2G2 and other two markers respectively (*P* = 0.68). We found a correlation between CK19-2G2 levels and CEA (*P*<0.001) or CA153 (*P*<0.001) levels in MBC patients but not in PBC patients. (*P* = 0.56, *P* = 0.93).

In addition, for cancer patients, CK19-2G2 levels in patients with MBC (Mean = 7.96 mU/ml, SE = 1.96 mU/ml) were higher than those with stage I-III PBC (Mean = 2.61 mU/ml, SE = 0.23 mU/ml) (*P* = 0.007) and DCIS (Mean = 1.85 mU/ml, SE = 0.39 mU/ml) (*P* = 0.002) respectively. There was also a significant differentiation in distribution of CK19-2G2 between stage I-III PBC and DCIS patients (*P* = 0.012). (Figure 2B).

### Relationship between Serum CK19-2G2 Levels and Clinicopathological Characteristics

We observed that high serum CK19-2G2 levels were significantly correlated with stage III (*P*<0.001 ), large size (≥2 cm) (*P = *0.020 ), positive lymph nodes (*P = *0.019 ), ER-negative status and HER2-positive status (*P = *0.006) (Table 2). Patients with positive lymph nodes, stage III and HER2 positive status had higher serum CK19-2G2 levels (2.23±7.62, 3.51±11.29, 3.09±11.06 mU/ml) than those with negative lymph nodes, stage I/II and ER positive status (1.11±2.32, 1.27±2.13, 1.38±2.33 mU/ml) (Figure 2B). However, there was no statistical difference in different size (*P = *0.306) and ER status (*P = *0.104). There were also no difference in serum CK19-2G2 levels according to age (*P* ***= ***0.355), menstruation status (*P = *0.542) and PR status (*P = *0.211).

**Table 2:** Association between serum cytokeratin19-2G2 and clinicopathological characteristics in PBC patients.

Characteristics	N	Serum CK19-2G2	levels (mU/mL)	*P* value
		<2	≥2	
Age (years)	249			0.355
≤35		21	6	
>35		192	30	
Menopausal status	249			0.542
Premenopausal		124	19	
Postmenopausal		89	17	
Stage	230			<0.001*
I, II		153	18	
III		41	18	
Tumor size (cm)	230			0.020*
≤2		64	5	
>2		124	30	
Lymphonodes status	245			0.019*
Negative		108	11	
Positive		101	25	
ER status	245			0.006*
Negative		72	21	
Positive		137	15	
PR status	245			0.118
Negative		87	24	
Positive		122	12	
CerbB2 status	234			0.005*
Negative		159	24	
Positive		49	12	

* *P* value <0.05, statistically significant.

### Impact of Serum CK19-2G2 on Prognosis in Primary Breast Cancer

With a median follow-up of 90 months, recurrence was observed in 51 patients (20.5%). 8 patients (15.7%) had locoregional recurrence and 43 patients (84.3%) had metastasis; supraclavicular lymph node involvement was observed in 6 patients and distant organs metastasis was observed in 37 patients. 48 patients died of recurrence by the observing end point of the 30^th^, December 2010.

**Figure 3:**
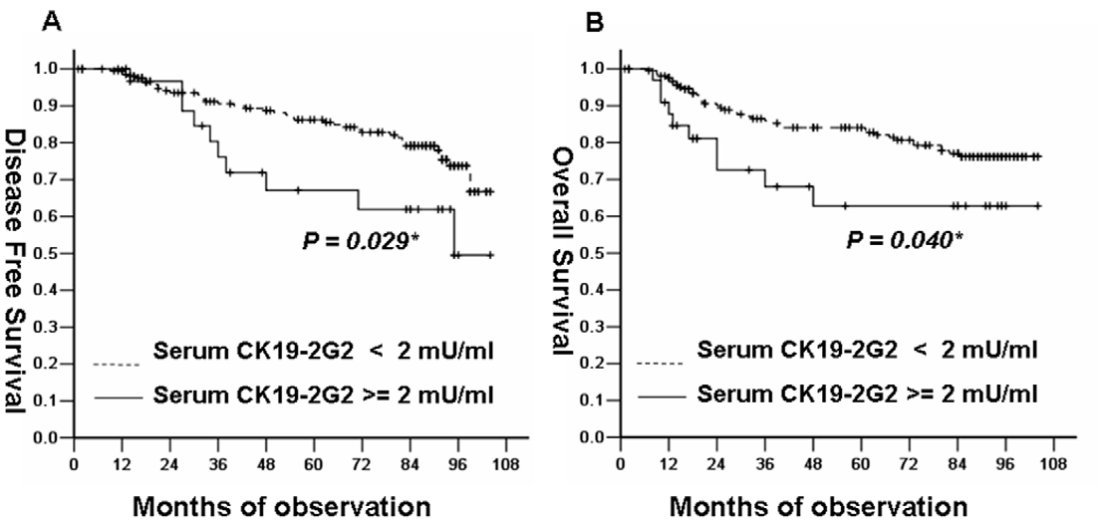
Survival curves for DFS and OS in breast cancer patients according to serum CK19-2G2 levels. (**A**) Kaplan-Meier survival curve showed that patients with high serum CK19-2G2 levels (≥2 mU/mL) had a significantly shorter disease free survival(*P = *0.029) than those with lower levels(<2 mU/mL). (**B**) Kaplan-Meier survival curve showed that patients with high serum CK19-2G2 levels (≥2 mU/mL) had a significantly shorter overall survival(*P = *0.040) than those with lower levels(<2 mU/mL).

There were 10 (28.6%) events in 35 patients with high serum CK19-2G2 levels and 41 events (19.4%) in 211 patients with low serum CK19-2G2 levels. Breast cancer patients with high serum CK19-2G2 levels (≥2 mU/mL) had a significantly shorter disease free survival(*P = *0.029) (Figure 3A) and overall survival (*P = *0.040) than those with lower levels (Figure 3B). A univariate analysis of survival showed that patients with post-menopasal, larger size (≥2 cm), positive lymph nodes, negative ER status, positive HER2 status and high serum CK19-2G2 levels had significantly shorter survival. Age and PR status did not appear to have any impact on prognosis (Table 3). Furthermore, significant variables found in univariate analysis were reanalyzed by multivariate analysis on prognosis. Unlike the results from univariate analysis, only serum CK19-2G2 as well as lymph nodes was showed to be an independent prognostic indicator.

**Table 3:** Cox regression analysis for variables considered for DFS and OS.

		DFS			OS	
	HR	95% CI	*P* value	HR	95% CI	*P* value
Univariate analysis						
Age	1.11	0.44–2.8	0.83	0.89	0.27–0.92	0.85
(≤35y versus >35y)						
Menopausal status	2.15	1.19–3.89	0.01*	2.12	1.09–4.11	0.03*
(post- versus pre-)						
ER status	2.38	1.18–4.82	0.02*	2.31	1.20–4.47	0.01*
(negative versus positive)						
PR status	1.51	0.81–2.73	0.17	1.69	0.88–3.26	0.12*
(negative versus positive)						
Tissue HER2 status	1.73	0.93–3.23	0.08	2.00	1.00–3.97	0.049*
(positive versus negative)						
Size	2.52	1.11–5.74	0.02*	2.07	0.89–4.80	0.09
(>2 cm versus ≤2 cm)						
Lymph nodesstatus	2.79	1.43–5.42	0.002*	2.74	1.32–5.68	0.007*
(positive versus negative)						
Stage	1.52	0.76–3.04	0.24	2.88	1.39–5.98	0.005*
(III versus I,II)						
Serum CK19-2G2	2.14	1.18–3.87	0.01*	2.88	1.39–5.98	0.005*
(high versus low)						
Multivariate analysis						
Serum CK19-2G2	3.74	1.74–8.05	0.001*	3.76	1.56–9.04	0.048*
(high versus low)						
Lymph nodes status	2.67	1.22–5.85	<0.001*	3.32	1.22–9.02	0.019*
(positive versus negative)						

* *P* value <0.05, statistically significant.

## Discussion

A variety of biological tumor markers for breast cancer have been investigated, such as CEA [12]–[14], CA 153[15]–[16], CA27.29 [17], [18], CA549 [19], Her2 [20]–[24], Cytokeratin8/18 [25], [26], CYFRA21-1[27]–[29], TPS [30]–[32], TPA [33] and other markers. However, to our knowledge, only CEA and CA153 are utilized frequently in clinical practice for breast cancer. Cytokeratin19-2G2 levels have been reported to be elevated in lung cancer and superior to CYFRA21-1 [11], another cytokeratin19 fragment, which has been considered as the most sensitive tumor marker for lung cancer. However, to our knowledge, no previous research reported of CK19-2G2 in breast cancer. In our study, we tested serum CK19-2G2 levels in four hundred and seventeen patients including three hundred and three (fifty-four DCIS and two hundred and forty-nine stage I-III) PBC patients and one hundred and fourteen MBC patients,eighty-one healthy controls and twenty-one breast benign disease patients. According to the data of our study, Serum CK19-2G2 appears not to be appropriate for screening or early diagnosis for breast cancer because of low sensitivity of CK19-2G2 for Stage 0/I and II diseases. With development of medical imaging, those tumors that could not be palpated by physical examination may be detected by mammography, ultrasound and MRI examination. Therefore, compared with other tumors, it is relatively easy to diagnose breast cancer at early stages due to the combination of palpation and imaging screening. Tumor makers, especially existed in the circulation, as the products of a disease developing to a certain stage, are speculated to be limited in detecting it at early stage but may be helpful in monitoring recurrence of the disease or judging the response to therapies including surgery, chemotherapy, radiation, endocrine and targeted therapy. In this study, serum CK19-2G2 showed a high sensitivity as almost the same as CA 153 in patients with Stage III and MBC. It could be observed that the sensitivity of CK19-2G2 was increasing with advanced stages. At the same time, serum CK19-2G2 levels of all healthy and benign controls are under the limit value, which indicates that its high specificity may lead to a good clinical utility.

Though ASCO recommend limited utility of CEA and CA 153 in clinical practice, elevated CA 153 and CEA levels are considered to be used to monitor response to therapy and indicate treatment failure when a readily measurable lesion is unavailable. We have demonstrated that the sensitivity of CK19-2G2 for breast cancer is as high as that of CA 153; consequently, the above tumor marker guidelines established by ASCO also might apply to CK19-2G2. However, we observed that the distributions for high serum CK19-2G2 levels were somewhat different from those for high serum CA 153 and CEA levels, that maybe caused by the reason that CK19-2G2 is one fragment of polypeptide while CA 153 and CEA are glycoproteins. At least CK19-2G2 can be a potentially useful tumor marker for breast cancer patients with negative CA 153 and negative CEA levels.

In our study, high serum CK19-2G2 levels were correlated with stage III (*P*<0.001), large size (≥2 cm) (*P* = 0.020), positive lymph nodes (*P* = 0.019), negative estrogen receptor (P = 0.006) and positive HER2 status (*P* = 0.005). Though no previous reports of serum CK19-2G2 in breast cancer, another CK19 fragment, CYFRA21-1, have been investigated and found that elevated serum CYFRA21-1 levels were correlated with advanced stage, large size and positive lymph nodes but no correlation with ER and PR status.

To our knowledge, there has not been any study exploring the prognostic value of serum CK19-2G2 levels in breast cancer patients. Bunzo Nakata et al reported the prognostic value of CYFRA21-1 for breast cancer, though they belong to Cytokeratin19, not the same fragment. The results from our study showed that patients with high CK19-2G2 levels have shorter disease free survival and overall survival. Furthermore, univariate and multivariate survival analysis demonstrated that serum CK19-2G2 levels are an independent factor for prognosis in breast cancer. Furthermore, considering that CK19-2G2 was found to be of higher sensitivity in MBC while relatively lower sensitivity in PBC, it seems not to be appropriate to be a diagnostic tumor marker for early detection but a candidate for monitoring recurrence in metastatic breast cancer.
